# Dual channel rank-based intensity weighting for quantitative co-localization of microscopy images

**DOI:** 10.1186/1471-2105-12-407

**Published:** 2011-10-21

**Authors:** Vasanth R Singan, Thouis R Jones, Kathleen M Curran, Jeremy C Simpson

**Affiliations:** 1 School of Medicine and Medical Science, University College Dublin, Belfield, Dublin 4, Ireland; 2 Imaging Platform, Broad Institute of MIT and Harvard, Cambridge, MA, USA; 3 Institut Curie, Departement du Transfert, 75248 Paris, France; 4 School of Biology and Environmental Science & Conway Institute of Biomolecular and Biomedical Research, University College Dublin, Belfield, Dublin 4, Ireland

**Keywords:** Quantitative co-localization, image analysis, non-parametric rank correlation, intensity weighting

## Abstract

**Background:**

Accurate quantitative co-localization is a key parameter in the context of understanding the spatial co-ordination of molecules and therefore their function in cells. Existing co-localization algorithms consider either the presence of co-occurring pixels or correlations of intensity in regions of interest. Depending on the image source, and the algorithm selected, the co-localization coefficients determined can be highly variable, and often inaccurate. Furthermore, this choice of whether co-occurrence or correlation is the best approach for quantifying co-localization remains controversial.

**Results:**

We have developed a novel algorithm to quantify co-localization that improves on and addresses the major shortcomings of existing co-localization measures. This algorithm uses a non-parametric ranking of pixel intensities in each channel, and the difference in ranks of co-localizing pixel positions in the two channels is used to weight the coefficient. This weighting is applied to co-occurring pixels thereby efficiently combining both co-occurrence and correlation. Tests with synthetic data sets show that the algorithm is sensitive to both co-occurrence and correlation at varying levels of intensity. Analysis of biological data sets demonstrate that this new algorithm offers high sensitivity, and that it is capable of detecting subtle changes in co-localization, exemplified by studies on a well characterized cargo protein that moves through the secretory pathway of cells.

**Conclusions:**

This algorithm provides a novel way to efficiently combine co-occurrence and correlation components in biological images, thereby generating an accurate measure of co-localization. This approach of rank weighting of intensities also eliminates the need for manual thresholding of the image, which is often a cause of error in co-localization quantification. We envisage that this tool will facilitate the quantitative analysis of a wide range of biological data sets, including high resolution confocal images, live cell time-lapse recordings, and high-throughput screening data sets.

## Background

The presence of a wide array of organelles enables eukaryotic cells to perform multiple and even competing biological processes in parallel. The cellular distribution of these organelles, and in particular the proteins and other molecules associated with them, remains of intense interest to the scientific community. Indeed the identification and understanding of the localization of all the proteins encoded by the genome can be considered as a first critical step towards assigning function [1]. Following the completion of various genome-sequencing projects many labs have been highly active in developing methodologies and tools to systematically assess the localization of the proteins encoded. Imaging-based technologies are particularly relevant to this task as they not only provide spatial information in a cellular context, but when applied in living cells can also provide information about protein dynamics over time. The first genome-wide assessment of protein localization was carried out in the yeast *Saccharomyces cerevisiae*, using a green fluorescent protein (GFP)-tagging approach [2]. Similar systematic approaches to reveal the localization of the human proteome have also been described [3,4], however this task remains to be completed. Apart from the sheer complexity of mammalian genomes and their extensive transcriptional products, systematic analysis of protein localization in higher eukaryotes is also hampered by a lack of software tools to aid in automated determination of localization. Some tools to automatically annotate localization have been reported [5], however due to the highly diverse morphology of the same organelles between different cells this approach remains challenging. One potential solution to this problem is to combine different fluorescently-labeled proteins (or fluorescently-labeled antibodies) in the same cells. Quantification of the abundance of a molecule, and its relative distribution compared to known organelle markers (co-localization), would provide a more accurate description of localization, and potentially could be applied on a genome-wide scale.

Co-localization, representing the co-compartmentalization of specific molecules, can be defined as the existence of spatial overlap between two molecules. The existence of overlap can be most simply determined by visual inspection of merged channels (although this is subjective and dependent on the expertise of the researcher). A second possibility is the use of a scatter plot - a 2D histogram representing the pixel intensities across two colour channels from a merged image. The component along the diagonal of this plot represents co-localized regions. This plot however assumes that the intensities across the two images are similar, which is not often the case, and therefore can produce aberrant results. A linear least-square fit of intensities of the two channels can be used to normalize for the differences, but this is not easily applicable to large numbers of diverse images.

The Pearson's correlation (PC) coefficient was the first described quantitative measurement approach for comparing dual channel images. This method gives one number that represents the overall correlation of intensities between two channels. The Pearson's correlation coefficient between two channels A and B is represented as follows:

$$\mathrm{PC}=\frac{\sum_{i}\left(A_{i}-A_{\mathrm{Avg}}\right)*\left(B_{i}-B_{\mathrm{Avg}}\right)}{\sqrt{\sum_{i}{\left(A_{i}-A_{\mathrm{Avg}}\right)}^{2}*{\left(B_{i}-B_{\mathrm{Avg}}\right)}^{2}}}$$

In this equation *A_i _*and *B_i _*are the intensities of pixel *i*, and *A_avg _*and *B_avg _*are the average intensities of channels A and B respectively. Although PC is used widely by the microscopy community to assess co-localization, the PC value generated is highly sensitive to the intensity in each channel. In microscopy images, the intensity values acquired from two channels can be highly different as a result of many factors, including nature of the organelle or protein under investigation, the brightness of the fluorophores, and the manner in which the images were generated. The high sensitivity of PC to channel intensities can therefore cause skewed results, and so awareness of this is vital.

A modification to address this deficiency in the original PC equation was formulated [6]. In this modified equation the intensity values in each pixel, without subtracting the average intensities in each channel, are applied. This new coefficient (r) is now expressed as:

$$r=\frac{\sum_{i}\left(A_{i}\right)*\left(B_{i}\right)}{\sqrt{\sum_{i}{\left(A_{i}\right)}^{2}*\sum_{i}{\left(B_{i}\right)}^{2}}}$$

In this formula *A_i _*and *B_i _*are the intensities of pixel *i *in channels A and B respectively. Manders and colleagues suggested dividing the coefficient into two components in order to cancel out the bias coming from the number of objects in each channel [6]. The overlap coefficients k_1 _and k_2 _provide a measurement of overlap between one channel and another, and are represented as:

$$k_{1}=\frac{\sum_{i}\left(A_{i}\right)*\left(B_{i}\right)}{\sqrt{\sum_{i}{\left(A_{i}\right)}^{2}}};\;k_{2}=\frac{\sum_{i}\left(A_{i}\right)*\left(B_{i}\right)}{\sqrt{\sum_{i}{\left(B_{i}\right)}^{2}}}$$

Manders went on to propose a new measure for co-localization based on the proportion of signal overlap, independent of the influence of pixel intensities. These coefficients, M_1 _and M_2 _are represented as:

$$M_{1}\frac{\sum_{i}A_{i,\mathrm{coloc}}}{\sum_{i}A_{i}};\;M_{2}\frac{\sum_{i}B_{i,\mathrm{coloc}}}{\sum_{i}B_{i}}$$

In this formula *A_i, coloc _= A_i _*if *B_i _> 0 *and *B_i, coloc _= B_i _*if *A_i _> 0*. One limitation of these co-localization equations is that thresholds first need to be manually identified in order to eliminate background and/or noise, and therefore this process can introduce bias. In this regard, Costes et al. demonstrated the importance of threshold for more accurate quantitative co-localization, and proposed an automated thresholding algorithm to define independent thresholds for each of the channels by pixel intensity correlations between the two channels [7]. To obtain the thresholds, each pixel in both the channels is represented as two components; a co-localized component and a random component (*A_i _= C + R1*, where *C *represents the co-localized component and *R *represents the random component). A stoichiometry constant α is introduced in the second channel of the co-localized component to account for the variability in co-localization ratio (Bi = α * C + R2). The thresholds for the two channels A and B are then set to *T *and a*T+b where *a *corresponds to the stoichiometry constant α, and *b *represents the mean random overlap difference between A and B after correction for the variability α. The thresholds are determined simultaneously for both channels by setting the thresholds at the highest intensity values and decreasing the thresholds *T *and a*T+b simultaneously on both channels until the PC of the remaining pixels below the thresholds is 0. The co-localization coefficients are represented as:

$$M_{1}C≅\frac{\sum_{i>T}A_{i}}{\sum_{i}A_{i}}\forall A_{i}>a*T+B$$

$$M_{2}C≅\frac{\sum_{i>AT+B}B_{i}}{\sum_{i}B_{i}}\forall B_{i}>T$$

One of the main drawbacks is the overestimation of co-localization quantified in these methods. In each of these cases, the contribution of a pixel to quantification of co-localization is binary, by classifying them as either 100% co-localized or 0% co-localized. In calculating the percentage of co-localization of the first channel with the second channel, while weighting is given to the intensity values in that first channel (Channel A for M1), the intensity value of the corresponding pixel position in the second channel (Channel B) is completely ignored. However, the intensity of the corresponding pixel position in the other channel indicates the relative abundance of the molecule of interest in that channel and therefore this should be accounted for. Differences in pixel intensities across the two channels render it difficult to combine these values and hence the classification is binary.

While PC estimates the overall correlation of intensities within a particular region of interest, it does not discriminate overlapping pixels from non-overlapping pixels. If the relative number of non-overlapping pixels is larger than the number of overlapping pixels, the correlation can be skewed by the intensity variation in the non-overlapping pixels. The Manders' coefficient is insensitive to intensity correlation and therefore the co-localization is quantified as a ratio of overlapping pixels to the total number of pixels. In order to address these deficiencies in the currently available co-localization algorithms, we have devised a new algorithm that not only takes account of correlating pixels between two channels, but also considers their relative intensities. We propose that this 'dual channel rank-based intensity weighting coefficient' (RWC) provides the most accurate measurement to date of co-localization between two image channels.

## Results and Discussion

### Rank-based intensity weighting

We propose a new weighting measure that overcomes the drawbacks described above, by weighting each pixel position in each channel based on the relative strength of intensities between the two channels. The uneven distribution of intensities between two channels warrants the use of a non-parametric approach for integrating these values. The weighting of co-localized pixels thus discriminates pixel positions with a similar intensity from those having extreme values. This ensures that co-localized pixel positions, where the two markers under investigation also have a high correlation of intensity, contribute more to the coefficient compared to poorly correlated positions. The new coefficients for each of the channels can be represented as rank-weighted co-localization coefficients RWC_1 _(amount of A co-localizing with B) and RWC_2 _(amount of B co-localizing with A) as follows:

$$\mathrm{RW}C_{1}≅\frac{\sum_{i=1}^{n}A_{i,\;\mathrm{coloc}}*W_{i}}{\sum_{i=1}^{n}A_{i}}\forall A_{i}>A_{\mathrm{Thr}}$$

$$\mathrm{RW}C_{2}≅\frac{\sum_{i=1}^{n}B_{i,\;\mathrm{coloc}}*W_{i}}{\sum_{i=1}^{n}B_{i}}\forall B_{i}>B_{\mathrm{Thr}}$$

In this formula weight $W_{i}=\frac{\left(\mathrm{Rn}-D_{i}\right)}{\mathrm{Rn}}$, A*_i, coloc _*= A*_i _*if B*_i _*> B*_Thr _*, 0 otherwise, and B*_i, coloc _*= B*_i _*if A*_i _*> A*_Thr _*, 0 otherwise. *Rn *is the maximum of ranks of channel A and B, whichever is the largest, and D is the absolute difference between the ranks of channel A and channel B for each pixel position *i *given by D*_i _*= **|**(Rank(A*_i_*) - Rank(B*_i_*)**|**. Parameters A*_Thr _*and B*_Thr _*are threshold values for channels A and B, respectively. The provision of defined threshold values is not necessary in this formula, as the ranking of pixels already discriminates low intensity pixels from high intensity pixels. However, by including these threshold parameters in the formula, users can still control the minimum pixel intensity above which co-localization quantification should be performed. Furthermore, it also allows easy comparison with other co-localization methods that require threshold information to be manually entered. If no manual intervention is required, the threshold values A*_Thr _*and B*_Thr _*are set to zero.

The ranking of pixels is made in each channel by giving the pixel(s) with the highest intensity a rank of 1 and assigning the next highest intensity pixel(s) a rank of 2, and so on. Pixels having the same intensities are assigned the same rank. The number of ranks in each channel depends on the number of grey levels in that channel. This method of ordinal ranking of pixels normalizes for the intensity values without altering the image. Each pixel in each channel gets a rank based on its intensity relative to the highest intensity in its channel. For an *n*-bit image, the ranks in each channel can range from 0 (for an image with no signal) to 2*^n ^*(for an image having all the possible grey levels). The number of ranks in each of the channels is determined, and '*Rn*' is assigned to the largest of these values.

The weighting for each pixel position is derived from the following expression, $\frac{\left(\mathrm{Rn}-D_{i}\right)}{\mathrm{Rn}}$. If a pixel position in each of the channels has the same rank, the expression will tend to 1, thereby the weight has minimal contribution to the co-localization coefficient. The further apart the ranks of the pixel position, the more the weight will tend to $\frac{1}{\mathrm{Rn}}$, and for extreme rank differences of which the maximum *D_i _*can be *Rn-1*, the weight will be $\frac{1}{\mathrm{Rn}}$. The weight can range from $\frac{1}{\mathrm{Rn}}$ to 1 corresponding to the maximum rank difference to the same rank, respectively. For an *n-*bit image, the maximum possible range is when all the grey levels are present in one of the two channels and this will range from $\frac{1}{2^{n}}$ to 1. The greater the number of grey levels present, the higher is the sensitivity and resolution of weighting. The sensitivity of weight depends on *Rn *and the sensitivity can be reduced by modifying the weight to $\frac{\left(Rn^{\prime }-D_{i}\right)}{Rn^{\prime }}$ where *Rn' *is a linear algebra equation derived from *Rn *such that Rn' = Rn + k*Rn, and *k *can take values from 0 to 1 and correspond to weights ranging from $\frac{1}{\mathrm{Rn}}$ to 1 for *k = 0 *and 0.5 to 1 for *k = 1*. The absolute difference between ranks ensures that the same weighting can be used for co-localizing pixel positions in both the channels and the weighting depends only on the difference of ranks. We envisage that this ranking approach could also be used for segmentation, for example to identify particular objects within an image based on a reference channel.

The weight represents the relative amount of co-localization and this can then be used for each pixel position to determine the degree of co-localization. Rank-based weighting addresses the critical issues of difference in channel illumination, dual channel directional illumination, and uniform noise and gradient correlation, as the ranks are preserved even though the actual intensities might suffer degradation in all of these cases. This method demonstrates a statistically efficient meta-analysis approach of combining both pixel co-occurrence and intensity correlation to improve co-localization analysis.

### Synthetic data sets

**Figure 1:**
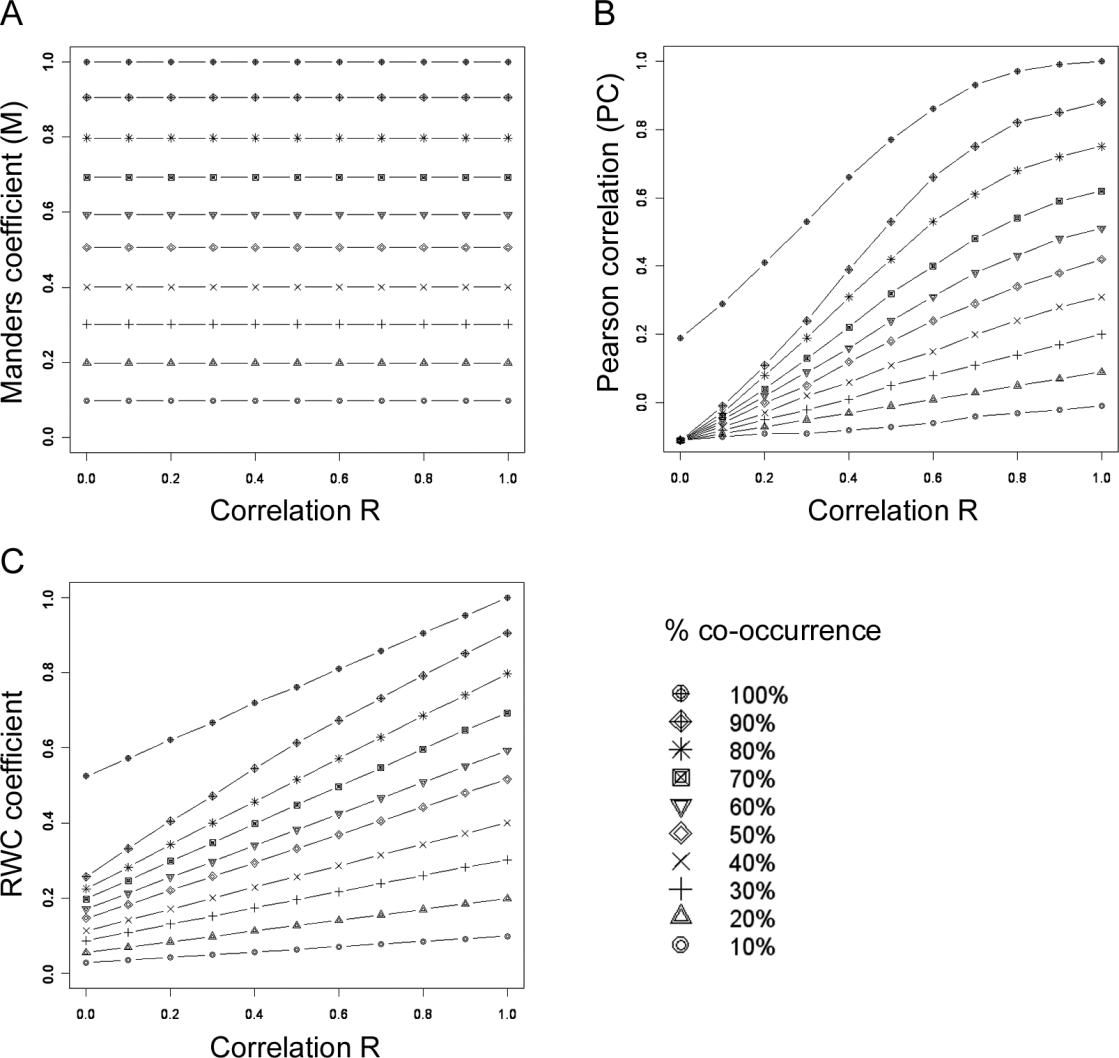
**Response of various co-localization algorithms to correlation and co-occurrence**. The sensitivity of various co-localization algorithms to varying levels of correlation and co-occurrence is tested. Sets of synthetic dual channel images with varying levels of co-occurrence and correlation are analyzed with Manders', Pearson's and RWC algorithms. (A) The Manders' co-localization coefficient is found to be insensitive to correlation. (B) Pearson correlation is insensitive to co-occurrence as shown by a poor linear response to varying levels of co-occurrence. The response is skewed as a result of correlation in the non-co-occurring pixels. (C) The RWC co-localization algorithm shows a linear response and is sensitive to both correlation and co-occurrence.

In order to test our algorithm we first designed a series of synthetic data sets. A pair of 256*256 8-bit images with pixel-sized objects was synthesized, having Gaussian distributions with a mean value of 128 and a standard deviation of 128. The correlation of the intensities of the overlapping pixels was then modified to generate a set of images having correlations ranging from R = 0 to R = +1. This set of images, containing both varying levels of co-occurrence and correlation, were tested with Manders, Pearson and RWC co-localization algorithms. As shown in Figure 1A, the Manders' coefficient was insensitive to the correlation of the pixel intensities. Similarly, as shown in Figure 1B, the Pearson correlation measurement was insensitive to co-occurring pixels and the response was skewed as a result of correlation seen in the non-co-occurring pixels. By contrast, the RWC approach was able to combine both co-occurrence and correlation information, thereby producing sensitive and meaningful co-localization coefficient (Figure 1C).

**Figure 2:**
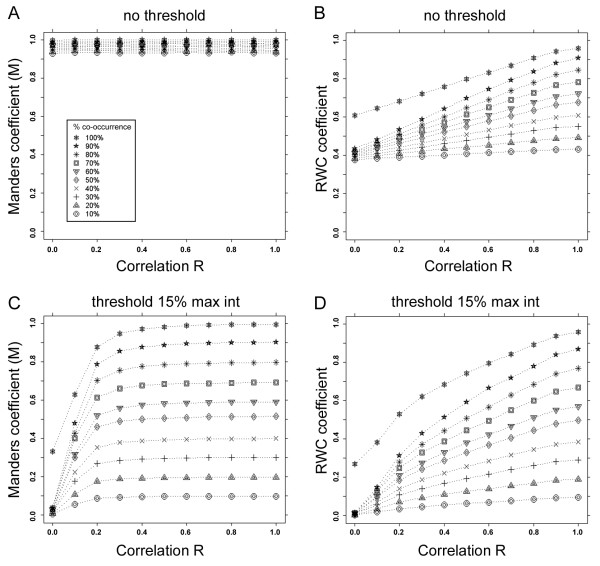
**Response of Manders' and RWC co-localization algorithms in the presence of noise**. The sensitivity of various co-localization algorithms to varying levels of correlation and co-occurrence is tested in the presence of noise (SD = 10). The same sets of synthetic data as used in Figure 1 were used, but with the addition of random noise having a distribution with standard deviation of 10. (A) With no thresholding, the Manders' co-localization coefficient at all instances of co-occurrence was found to be greater than 0.9. (B) With no thresholding, the RWC co-localization coefficient displayed a reduced dynamic range, although the response to co-occurrence was still linear. (C) With thresholding at 15% of the maximum intensity the Manders' co-localization coefficient was insensitive to correlation. At higher levels of correlation the thresholding eliminated the effects of noise, however at low correlation values the Manders' coefficient was still sensitive to noise. (D) With thresholding at 15% of the maximum intensity RWC showed a good response to both co-occurrence and correlation, as the threshold suppressed the effects of the noise.

In order to further validate the robustness of our algorithm we modified the synthetic data used in Figure 1 to include random noise, having a normal distribution with standard deviation of 10. We first compared the response of Manders' and RWC coefficients in the presence of this noise (Figure 2). Strikingly, when the images were not subjected to thresholding (as in Figure 1) the noise had a much greater effect on the Manders' coefficients (Figure 2A) compared to the RWC coefficients (Figure 2B). Although the dynamic range of the RWC coefficients was reduced, the coefficients observed still showed a linear response to varying degrees of co-occurrence. We next introduced a threshold (at 15% of maximum intensity values in each channel) in order to potentially suppress the effect of the noise. These experiments revealed that the response curves of both the Manders' and RWC co-localization coefficients returned to similar profiles to those shown in Figure 1 (Figure 2C and 2D), with the exception that at lower levels of correlation (R < 0.2) the noise effect was still visible.

**Figure 3:**
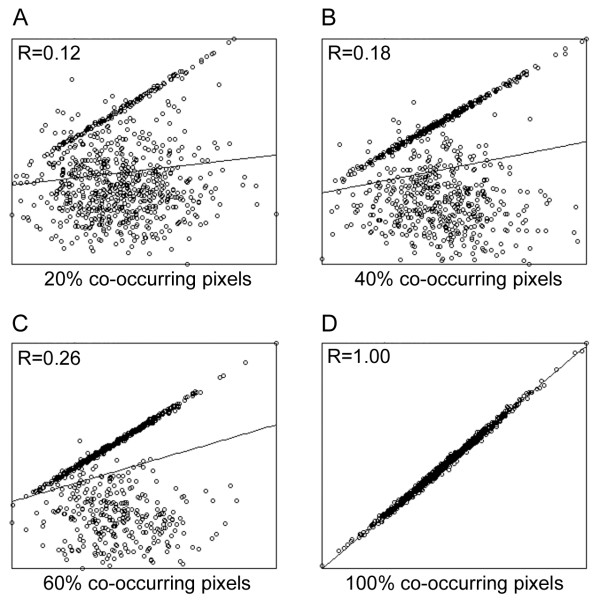
**Effect of non-co-occurring pixels on correlation**. Sets of synthetic dual channel images with varying levels of co-occurrence were generated with all the co-occurring pixels in the image having a correlation of R = 1.00, and all the non-occurring pixels in the image having a correlation of R≈0.00. (A) Example showing the presence of 20% co-occurring pixels, resulting in an overall correlation of R = 0.12 for the entire image. (B) Example showing the presence of 40% co-occurring pixels, resulting in an overall correlation of R = 0.18 for the entire image. (C) Example showing the presence of 60% co-occurring pixels, resulting in an overall correlation of R = 0.26 for the entire image. (D) Example showing the presence of 100% co-occurring pixels, resulting in an overall correlation of R = 1.00 for the entire image. This demonstrates the importance of using only the co-occurring pixels for quantifying correlation and co-localization.

We next determined the influence of non-co-occurring (non-overlapping) pixels on co-localization. As shown in Figure 3, the overall correlation can be negatively influenced by the presence of uncorrelated non-co-occurring pixels. Using a scenario in which only 20% of the pixels co-occur (Figure 3A), and where the correlation of the co-occurring pixels is 100% (R = 1), the overall correlation (including the 80% un-correlated non-co-occurring pixels) was found to be very low (R = 0.12). Although increasing the amount of co-occurring pixels to 40% and 60% (Figures 3B and 3C respectively) improves the overall correlation scores (R = 0.18 and R = 0.26 respectively), the uncorrelated non-co-occurring pixels still severely distort the overall correlation value. For this reason, it is important to consider the correlation of only co-occurring pixels in isolation. Indeed, when only co-occurring pixels were considered, the correlation was correctly determined (R = 1.0) (Figure 3D). It is also for this reason that a non-linear response was seen in Figure 1B and 1C.

**Figure 4:**
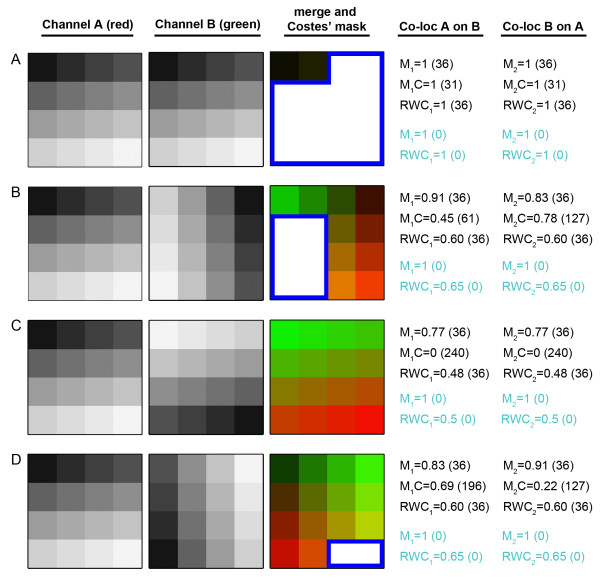
**Effect of intensity correlations on various co-localization algorithms**. A synthetic image composed of a 16 segment sub-grid, each of different intensity, was generated (channel A). The same image was duplicated and rotated at various angles (channel B). These images were analyzed by Manders' (M), Costes' (MC) and RWC algorithms. In the merged images, the Costes' mask (considered for co-localization analysis) is shown in white with a blue border. The remaining regions of the images are shown in their corresponding LUTs. The co-localization coefficients, and thresholds used (in parentheses) are also given. For calculation of the Manders' and RWC coefficients, the threshold was set at either 15% of the maximum intensity in each channel (black text), or no thresholds were applied (cyan text). (A) Two channels containing identical data result in co-localization coefficients of 1.0 in all cases. (B) The Costes' mask disregarded 62.5% of the image, despite the presence of high pixel intensities in these areas which should contribute to the overall quantification. (C) The Costes' mask disregarded the entire image because the intensities across the image were anti-correlating between the two channels. (D) The Costes' mask disregarded 87.5% of the image due to the varying correlation of intensities between the two channels. In all examples the RWC coefficients were as expected, based on the intensity distribution of the synthetic images. When no thresholding was applied the Manders' algorithm reported co-localizations coefficients of 1.0, whereas the RWC coefficients remained similar to those observed when thresholding.

A second set of 512*512 8-bit images (256 grey levels) was next synthesized, with each image composed of a 16 segment sub-grid (128*128 pixels) each of different intensity (Figure 4). In these images black pixels were assigned a grey value of 0, and white pixels a value of 255. The first (upper left) segment in each image was assigned an intensity value of 15, the next segment a value of 30, progressively adding 15 grey levels to each segment such that the final (lower right) segment had an intensity of 240. The original image was designated as 'channel A', and the rotation of this image sequentially by 90, 180 and 270 degrees was used to form the images for 'channel B'. Using these data, four co-localization experiments were performed, allowing us to analyze the effect of pixel intensity distribution across pairs of images with respect to co-localization. The third column shows the Costes' mask generated, based on the threshold set by Costes' automated threshold algorithm [7]. In the mask, co-localized pixels above threshold are shown in white (surrounded by a blue border), and other pixels are shown as a merge of their corresponding LUT, assigning channels A to red and B to green. The Costes' automated thresholding was performed using the JACOP plugin within ImageJ [8].

Applying the Costes' mask to the data in Figure 4 allowed us to determine that the proportion of pixels above the threshold was 87.5%, 37.5%, 0% and 12.5% respectively in each of the co-localization experiments (Figures 4A, B, C and 4D). We first analyzed the co-localization experiment in which the two channels were identical (Figure 4A). As expected, applying Costes' automated threshold resulted in 12.5% of the pixels being discarded from the mask, however because the images were perfectly correlating, the co-localization coefficients (M_1_C and M_2_C) were correctly calculated at 1.0 (Figure 4A). By contrast, in co-localization experiments in which the intensities did not correlate (Figures 4B, C and 4D), the majority of the pixels were discarded from the mask leading to incorrect co-localization coefficients. This was particularly striking in cases where there were pixels co-occurring in both channels, but anti-correlation of the intensities resulted in failure of the automated thresholding, in turn producing co-localization coefficients (M_1_C and M_2_C) of zero (Figure 4C). This scenario is especially relevant in biological samples where two molecules could have anti-correlating intensities, despite co-occurring. Applying the RWC algorithm to the synthetic data set in Figure 4 produced more realistic co-localization coefficients. This was because the algorithm considers both co-occurrence (specified by the threshold) and correlation. Even in the example of anti-correlating pixels, the RWC approach produces co-localization coefficients (RWC_1 _and RWC_2_) of 0.48, which are more representative of the intensity distribution (Figure 4C). Next we examined this synthetic data set without applying a threshold. Strikingly, in all the four cases, the Manders' algorithm always reported a co-localization coefficient of 1.0, whereas RWC produced similar values to those observed when the images had been thresholded. This highlights that the application of the Manders' algorithm always requires the application of careful thresholding, but that RWC is not sensitive to this requirement as it produces meaningful co-localization coefficients in the absence of thresholding. Use of the RWC methodology therefore eliminates the need for thresholding, which can be a source of significant bias when analyzing image data.

As a final test of the algorithm we subjected this synthetic data set to varying degrees of random noise, with standard deviations of 5, 10 and 15 (Table 1). Although RWC takes account of pixel intensity, we observed that even in the presence of very high levels of noise (SD = 15), the effects on the co-localization coefficients were negligible when appropriately thresholded (15% of maximum pixel intensity), except in the specific case of 100% co-occurrence and 100% correlation, where the coefficients became mildly distorted (Table 1, row 4A). This shows that RWC is comparable to existing co-localization methods in terms of its response to image noise.

**Table 1:** Effect of random noise on Manders and RWC co-localization coefficients

	Noise SD 5		Noise SD 10		Noise SD 15	
**FIGURE**	**Co-loc A on B**	**Co-loc B on A**	**Co-loc A on B**	**Co-loc B on A**	**Co-loc A on B**	**Co-loc B on A**

**4A**	M_1 _= 1.0	M_2 _= 1.0	M_1 _= 0.99	M_2 _= 0.99	M_1 _= 0.99	M_2 _= 0.99
	RWC_1 _= 0.98	RWC_2 _= 0.98	RWC_1 _= 0.95	RWC_2 _= 0.95	RWC_1 _= 0.92	RWC_2 _= 0.92

**4B**	M_1 _= 0.90	M_2 _= 0.83	M_1 _= 0.88	M_2 _= 0.84	M_1 _= 0.89	M_2 _= 0.85
	RWC_1 _= 0.62	RWC_2 _= 0.61	RWC_1 _= 0.62	RWC_2 _= 0.61	RWC_1 _= 0.61	RWC_2 _= 0.61

**4C**	M_1 _= 0.76	M_2 _= 0.76	M_1 _= 0.76	M_2 _= 0.76	M_1 _= 0.77	M_2 _= 0.77
	RWC_1 _= 0.5	RWC_2 _= 0.5	RWC_1 _= 0.49	RWC_2 _= 0.49	RWC_1 _= 0.49	RWC_2 _= 0.49

**4D**	M_1 _= 0.83	M_2 _= 0.90	M_1 _= 0.84	M_2 _= 0.88	M_1 _= 0.85	M_2 _= 0.89
	RWC_1 _= 0.61	RWC_2 _= 0.62	RWC_1 _= 0.61	RWC_2 _= 0.62	RWC_1 _= 0.61	RWC_2 _= 0.61

### Biological data sets

**Figure 5:**
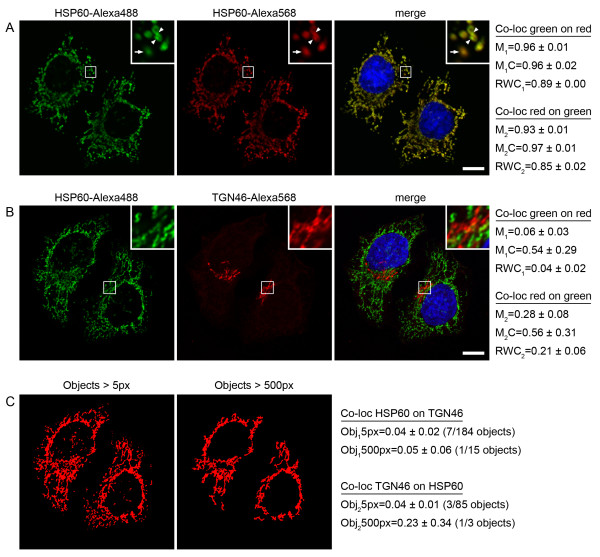
**Quantitative co-localization in immunostained cells**. HeLa cells were immunostained with primary antibodies against the mitochondrial protein HSP60 and the TGN protein TGN46, followed by fluorescently-labeled secondary antibodies as indicated. (A) Primary anti-HSP60 antibodies were detected with a cocktail of two secondary antibodies, labeled with either Alexa488 or Alexa568. Co-localization analyses were carried out and the results are shown. The inset shows a four-fold magnification of the marked area, with arrows indicating membranes displaying more intense red labeling, and arrowheads indicating membranes displaying more intense green labeling. The RWC algorithm was able to detect these subtle differences with a higher sensitivity. (B) Cells were double immunostained with primary anti-HSP60 and anti-TGN46 antibodies and detected with secondary antibodies labeled with Alexa488 and Alexa568 respectively. Co-localization analyses were carried out and the results are shown. The inset shows a four-fold magnification of the marked area. Bars represent 10 μm. (C) Objects detected at 5 pixel and 500 pixel minimum sizes from the combined HSP60 and TGN46 images shown in panel B. Obj5px and Obj500px represent the mean co-localization coefficients (defined from the number of co-localizing objects as a proportion of the total number of objects) from the test set of images analyzed, at minimum object sizes of 5 pixels and 500 pixels respectively. The threshold of object size used has a significant effect on the co-localization coefficient determined.

We next tested our co-localization algorithm on biological data. In the cellular context it is essential that we are able to discriminate the localization of proteins between different cellular components. This is particularly important with respect to membrane-bounded compartments, which occupy a significant volume within cells, can be very closely opposed to one another, but which carry out very different functions. In order to test the sensitivity of our algorithm we first probed cultured HeLa cells with a primary antibody directed against the mitochondrial chaperone HSP60. We then used a cocktail of two fluorescently labeled (Alexa488 and Alexa568) secondary antibodies to detect the primary antibody, and thereby produce a two channel image in which the same subcellular structures were labeled with two different fluorophores. Confocal imaging of these immunostained cells revealed, as expected, a very high degree of apparent co-localization between the two colour channels (Figure 5A). On application of the Manders' and Costes' algorithms we determined that the co-localization coefficients were on average 0.95 and 0.96, respectively. By contrast, analysis of the same image set using the RWC algorithm revealed a lower average co-localization coefficient of 0.87. Closer examination of the images revealed that the two secondary antibodies did not in fact evenly decorate the primary antibody, and it was possible to discern specific membrane elements that were more strongly labeled with either the Alexa488 or Alexa568 antibodies (Figure 5A, inset). These results indicate that as a consequence of the RWC algorithm considering the pixel intensity at each position within an image, it is able to discriminate very subtle differences between localization profiles.

We next performed a co-localization experiment with two different primary antibodies that recognize different membranes within the cell, specifically the chaperone HSP60 representing the mitochondria, and the putative cargo receptor TGN46 that has a steady-state localization at the *trans*-Golgi network (TGN) [9]. Confocal microscopy analysis revealed that both the Manders' and RWC algorithms could accurately discriminate these different membranes and produce low co-localization coefficients (Figure 5B). By contrast the co-localization coefficient determined by the Costes' algorithm performed very poorly, most likely as a result of the way it determines thresholds based on intensity correlations.

Rather than considering individual pixel intensities within an image, an alternative method of probing co-localization is to use object-based analysis [8]. This approach relies on the ability to discriminate and segment defined objects (of similar pixel intensity). Typically the centroid of each object is used as a reference point for comparison between the channels, and the number of co-localizing objects, as a fraction of the total number of objects detected, defines the degree of co-localization. We applied such a method to our HSP60-TGN46 biological data (as used in Figure 5B) using the JACOP plugin within ImageJ [8]. Applying the same thresholds as used previously, this analysis revealed vastly differing co-localization values for the same set of images, depending on the minimum pixel size used to determine objects. For example, at a minimum value of 5 pixels, 85 TGN46 objects were identified, of which only 3 co-localized with HSP60 (Figure 5C). However, increasing the minimum pixel size to 500 pixels, resulted in the detection of only 3 discrete objects, of which only 1 co-localized with HSP60. The consequence of this large discrepancy in the numbers of objects identified resulted in an overall change in object-based co-localization co-efficient from 0.04 to 0.23 for the test set of images analyzed. This indicates that while object-based co-localization methods can produce coefficients similar to co-occurrence methods, they are wholly dependent on the object segmentation and identification parameters given by the user. Moreover, the pixel intensity information is only used in the segmentation process rather than for quantification of co-localization, meaning that this valuable information is effectively discarded.

**Figure 6:**
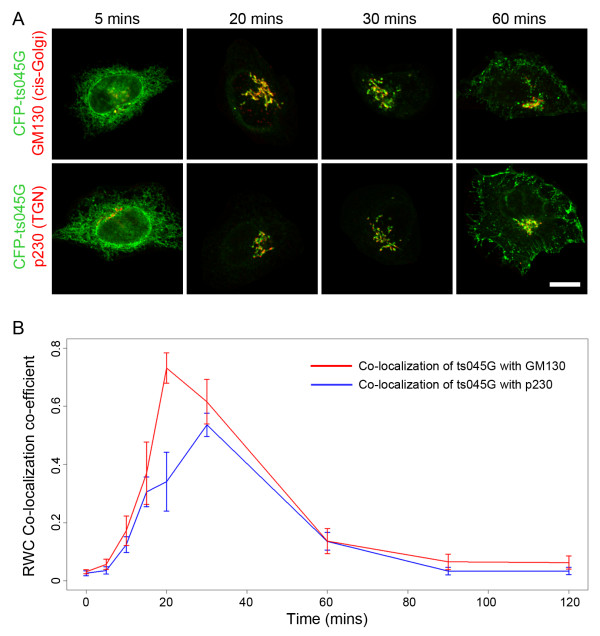
**Quantitative co-localization analysis of transport through the early secretory pathway**. HeLa cells were transfected with plasmids encoding the secretory cargo CFP-ts045G. Following incubation at the restrictive temperature for ts045G release from the endoplasmic reticulum, the temperature was reduced to allow folding and release of the cargo into the secretory pathway. Cells were fixed at various time points, immunostained for markers of the *cis*-Golgi complex (GM130) or the TGN (p230), and co-localization analyzed using the RWC algorithm. (A) Example images from various time points of release showing CFP-ts045G (green) moving from the endoplasmic reticulum (5 mins), through the Golgi complex (20 and 30 mins), and beginning to arrive at the cell surface (60 mins). Bar represents 10 μm. (B) RWC co-localization analysis of ts045G with GM130 and p230 demonstrated an initial peak of the cargo with the *cis*-Golgi marker at 20 mins, followed by a peak in co-localization with the TGN marker at 30 mins. The RWC algorithm was sufficiently sensitive to record this subtle change in localization. Error bars indicate mean and standard deviations between the 10 cells analyzed for each time point.

Finally we sought to test our algorithm in the context of a well established cellular assay that traditionally has required a biochemical approach for evaluation. Within cells the secretory pathway serves to transport proteins and lipids from their site of synthesis in the endoplasmic reticulum (ER), through the Golgi complex, ultimately out to the endosomal/lysosomal system or the cell surface. Characterization of the initial steps in this pathway (from ER to the *trans*-face of the Golgi complex) has largely been studied by following the change in glycosylation pattern of a temperature-sensitive viral glycoprotein (ts045G) [10] as it tracks through these compartments [11]. Imaging approaches to follow this model cargo molecule in living cells were first described in the late 1990s [12,13], however to date no quantitative co-localization-based approach to follow ts045G through the early secretory pathway has been reported. We therefore transfected HeLa cells with plasmids encoding a fusion of ts045G with the cyan fluorescent protein (CFP), and accumulated this cargo in the ER before releasing a wave of it into the secretory pathway. We then fixed cells at various time points after ER release, and carried out immunostainings with antibodies targeting the *cis*-Golgi marker GM130 or the TGN marker p230. Confocal images from each time point of the assay were acquired (Figure 6A), and RWC analysis was applied to determine the co-localization profile of ts045G with the *cis*- and *trans*-Golgi markers (Figure 6B). RWC co-localization analysis revealed a peak in co-localization of ts045G with the *cis*-marker after 20 minutes, followed by a peak with the *trans*-marker after 30 minutes (Figure 6B). Visual inspection of the images also revealed that the majority of ts045G had exited the Golgi complex after 60 minutes, and this was in good agreement with the low RWC coefficient determined at this time point. Overall, the kinetics of ts045G transit through the early secretory pathway, as measured by co-localization analysis, was extremely similar to that determined by biochemical techniques. The results from this assay clearly demonstrate the sensitivity of the RWC algorithm, as it was successfully able to discriminate co-localization at the entry and exit faces of a single organelle. Of particular interest are the time points 20 minutes and 30 minutes after ts045G release from the ER. At 20 minutes the majority of ts045G had arrived at the *cis*-face of the Golgi complex (high co-localization with GM130), but after this time the RWC algorithm was able to detect loss of the cargo from this side of the Golgi complex and accumulation at the *trans*-face of the organelle (high co-localization with p230). These measurements clearly demonstrate that this algorithm has the capacity to detect relatively small spatial changes in the distribution of proteins across a compact structure such as the Golgi complex. Furthermore, a co-localization-based approach not only has the advantage of being easier to perform than the equivalent biochemical technique, but also it provides quantitative data at a single cell level, therefore potentially making it suitable for high-throughput approaches.

## Conclusions

In this work we present a novel tool to precisely quantify co-localization between structures within biological images. Although a number of co-localization algorithms have been described previously, this is the first example of such a tool that takes account of both co-occurrence and correlation of pixels, combining them efficiently to produce a meaningful coefficient value. We demonstrate in this work, using both synthetic and biological data sets, that this algorithm is a robust tool that works effectively across a very wide range of situations, and that it eliminates the need for manual thresholding of images, which is a well established cause of error in co-localization analyses. We envisage that this tool will facilitate the quantitative analysis of a wide range of biological data sets, including high resolution confocal images, live cell time-lapse recordings, and high-throughput screening data sets.

## Methods

### Synthetic Data

Synthetic data sets used for Figures 1 to 3 were generated using the Image Processing Toolbox in MATLAB (MathWorks). A pair of 256*256 8-bit images with pixel sized objects were synthesized as a combination of a mask (foreground, FG) and intensity (I) having Gaussian distributions with a mean value of 128 and a standard deviation of 128. The masks for the two images, FG1 and FG2 did not overlap. A series of paired images were generated from the initial set by copying portions of FG1 on to FG2 to create varying levels of co-occurrence ranging from 10% to 100%. The correlation of the intensities of the overlapping pixels was then modified to generate a set of images having correlations ranging from R = 0 to R = +1, as described in [14]. Briefly, in order to obtain pairs of images with varying correlations, the intensities in one of the pair of the original images was replaced with the fraction of intensity obtained from the formula given below, allowing us to generate a set of images. The copy fraction C*_f _*is used to control the percentage of correlation between the pairs of images as indicated in the formula below.

$$A_{new}=A_{\mathrm{Avg}}+\left(A_{i}-A_{\mathrm{Avg}}\right)\left(1-C_{f}\right)+\left(B_{i}-B_{\mathrm{Avg}}\right)\left(C_{f}\right)$$

In this formula, A*_i _*and B*_i _*are the intensities of pixels at position *i *in channels A and B respectively in the initial pair of images. A*_new _*is the new intensity of channel A at position *i *for the new image. Varying C*_f _*from 0 to 1, with a step size of 0.1, generates correlations ranging from 0% to 100% (in 10% increments), between new image A and the initial image B. To introduce noise we used the 'Add Specific Noise' routine within ImageJ, setting this at 5, 10 and 15 standard deviations for various test cases.

### Cell Culture, Transfection and Immunostaining

HeLa cells were routinely cultured in Dulbecco's Modified Eagle's Medium (DMEM) supplemented with 10% foetal bovine serum (FBS) and 1% L-Glutamine at 37°C in a 5% CO_2 _incubator. Experiments were carried out on cells growing on glass coverslips maintained in 12-well plates. All transient transfections were performed using Fugene6 according to the manufacturer's instructions. The CFP-ts045G construct has been described previously [15]. Cells were fixed with either methanol or 3% PFA and quenched with 30 mM glycine. Primary mouse anti-HSP60 antibodies (BD Biosciences) sheep anti-TGN46 antibodies (Biozol), and mouse anti-p230 antibodies (BD Biosciences) were used. Secondary antibodies anti-mouse Alexa488 and Alexa568, and anti-sheep Alexa568 (Molecular Probes) were used for visualization. Coverslips were mounted on glass slides with Mowiol.

### Image Acquisition and Analysis

Confocal images were acquired with an Olympus FV1000 system using a 60x/NA1.35 oil immersion objective. Images were acquired at a resolution of 1024*1024 pixels, a pixel dwell time of 12.5 μs, and a 2.5-fold zoom. Sequential acquisition mode was used in all cases. Individual cells from each field of view were manually segmented, but not subjected to background correction or any further manipulation. A minimum of 10 cells were used for quantification of each time point and immunostaining. The Rank Weight Co-localization Coefficient (RWC) was implemented in ImageJ.

### Ts045G Assay

HeLa cells cultured on coverslips were transfected with plasmids encoding CFP-ts045G and incubated at 39.5°C for 12 h to accumulate the ts045G in the ER. Following this incubation cycloheximide (100 μg/ml) was added to prevent further protein synthesis. The temperature was lowered to 32°C to allow folding and release of ts045G from the ER. Coverslips were removed from this incubation at various time points and fixed in 3% PFA. Prior to immunostaining the cells were permeabilized with 0.1% Triton X-100 and then incubated with anti-GM130 or anti-p230 antibodies followed by incubation with secondary antibodies as described above.

## Authors' contributions

VRS devised the RWC algorithm, carried out the imaging experiments, performed the analyses, and helped to draft the manuscript. TRJ and KMC participated in the design and execution of the analyses. JCS conceived the study, participated in its design and coordination, carried out the imaging experiments and prepared the manuscript. All authors read and approved the final manuscript.
